# Differential diagnosis of thrombosis and myxoma in unusual heart positions by dual-energy CT multiple parameter imaging: A case series

**DOI:** 10.1097/MD.0000000000041303

**Published:** 2025-01-24

**Authors:** Yunting Wang, Yuxin Cui, Yunbing Chen, Honglin Lan

**Affiliations:** a Changzhi Medical College, Shanxi, China; b Department of Medical Imaging, Jincheng People’s Hospital, Shanxi, China.

**Keywords:** cardiac mass, dual-energy CT, Effective Atomic Number diagram, iodine density map, spectrum curve slope

## Abstract

**Rationale:**

Thrombus is the most common occupying lesion in the cardiac chambers, it is often distinguished from cardiac neoplastic occupations. Among them, the most common is cardiac myxoma, whose imaging manifestations are often confused with thrombus. However, the 2 types of lesions have different therapeutic strategies and are both potentially high-risk sources of embolism, so early differentiation between intracardiac thrombus and cardiac tumor is essential. In this study, we intend to investigate the value of dual-energy computed tomography (CT) in the differential diagnosis of cardiac thrombus and myxoma by retrospectively analyzing the dual-energy CT-related parameters of 2 cases of intracardiac thrombus and 1 case of cardiac myxoma.

**Patient concerns:**

Three cases of masses located in uncommon areas of the heart with comparable imaging characteristics are presented in this study.

**Diagnoses:**

Echocardiography revealed an isoechoic mass in the cardiac chambers, while CT scans showed a hypodense occupancy with varying morphologies. Postoperative pathology or follow-up after treatment confirmed 1 case as a right ventricular thrombus, another as a right atrial thrombus, and the third as a right ventricular myxoma.

**Interventions:**

In this study, we conducted a retrospective analysis of dual-energy CT-related parameters in 2 cases of intracardiac thrombus and 1 case of cardiac myxoma.

**Outcomes:**

Our findings indicate notable differences in the slopes of the energy spectral curves, mean iodine density, and effective atomic number between the intracardiac thrombus and myxoma cases.

**Lessons:**

Drawing upon existing literature, we propose combining different quantitative analysis methodologies to create a more objective foundation for distinguishing between cardiac thrombosis and myxoma.

## 1. Introduction

Cardiac thrombus is the most common occupying lesion in the heart, frequently occurring in the left atrium and left ventricle, and is associated with slow blood flow and blood stagnation in the cardiac chambers; thrombosis is rarely found in the right heart, and is most often caused by migratory thrombi in the deep venous system of the lower extremities.^[1]^ Cardiac myxoma are the most common benign tumors among primary cardiac tumors in adults, originating from subendocardial mesenchymal cells. They can arise in either heart chamber, with the majority occurring in the left atrium; cardiac myxoma originating in the right ventricle are extremely rare.^[2,3]^ In our three-case studies, all 3 cardiac cases were situated in the right heart. Cardiac thrombosis and myxoma, due to their distinct location and tissue structure, can both detach in response to high-speed blood flow in the heart cavity, resulting in acute embolism in systemic or pulmonary circulation and increasing patient mortality.^[4]^ Therefore, early imaging diagnosis is particularly critical.

Here, we report a series of cases that we retrospectively analyzed. These patients, 2 male and one female, had undergone echocardiography and dual-energy CT enhancement scanning prior to surgery or treatment. They had been diagnosed with one right ventricular thrombus, one right atrial thrombus, and one right ventricular myxoma, all of which were confirmed by postoperative pathology or treated thereafter, between 2021 and 2023. By examining the pertinent dual-energy CT characteristics, the utility of the imaging modality in the differential diagnosis of cardiac thrombus and myxoma was investigated.

The scanning approach is introduced as follow. The patient is placed in a supine position, and the chest breath holding localization image was first obtained. The contrast agent tracking automatic trigger mass injection mode was applied, and the scanning range was from the tracheal protrusion to 10 to 20 mm below the diaphragmatic surface of the heart. The scanning parameters are set up as follows. The voltage of tube A and tube B are 100 kV and Sn150 kV respectively, with adaptive tube current, detector collimation of 192 × 0.6 mm, reconstruction layer thickness of 0.75 mm, and reconstruction layer spacing of 0.5 mm. After scanning, Siemens syngo.via post-processing workstation was employed for image processing.

## 2. Case presentation

This study was approved by the Institutional Review Board of Jincheng People’s Hospital. (JCPH.NO20240223003). All the participants provided written informed consent.

General data, dual-energy CT parameters, and pathological findings of 3 patients are shown in Table 1.

**Table 1:** General data, dual-energy CT parameters, and pathological findings of 3 patients

No	Age	Lesion location	Lesion shape	Calcification	Iodine density (mg/mL)	Effective atomic number (HU/Z)	Spectrum curve slope	Lesion properties
1	51	RV	Striped	(−)	0.3	8.34	1.6	Thrombus
2	39	RA	Quasi-circular	(−)	0.6	8.15	1.1	Thrombus
3	67	RV	Quasi-circular	(+)	2.8	9.10	2.9	Myxoma

### 2.1. Case 1

**Figure 1.**
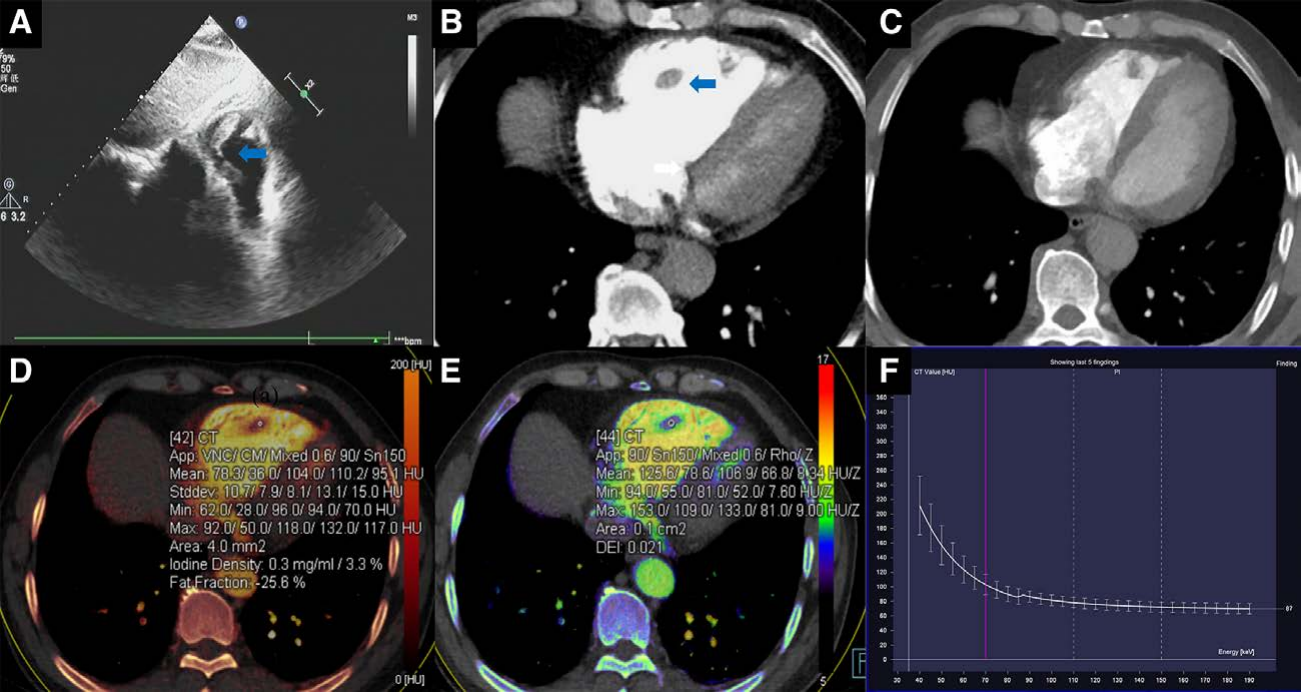
Case 1. The images show a thrombus in the right ventricle. Echocardiography, showed a striated mass in the right ventricle (A); CT enhancement scan, the right ventricle can be seen classified round filling defect (B); arterial iodine map, the patient’s lesion arterial iodine concentration of 0.3 mg/mL (D); the effective atomic number map, the patient’s lesion Zeff is 8.34 HU/Z (E); the energy spectral curve schematic diagram, the value of λ_Hu_ is 1.6 (F). Improved cardiac function, after anticoagulation, symptomatic treatment for 15 days and follow-up examination CTA of pulmonary artery: CTA of pulmonary artery did not show any abnormality, and there was no obvious abnormal density shadow in the right ventricle (C).

The patient, a 51-year-old man, received dual-energy CT and echocardiography scan after being taken to the hospital for 5 days with severe shortness of breath and chest pressure. The results of the echocardiography revealed a striated mass in the right ventricle (Fig. 1A); the CT enhancement scan showed a classified round filling defect in the right ventricle (Fig. 1B); the arterial iodine map showed a 0.3 mg/ml concentration of the patient’s lesion (Fig. 1D); the effective atomic number map revealed that the patient’s lesion Z effective (Zeff) is 8.34HU/Z (Fig. 1E); and the energy spectral curve schematic diagram showed that the value of λHu is 1.6 (Fig. 1F). The heart function was improved following anticoagulation and symptomatic therapy for 15 days, as well as a follow-up assessment Pulmonary artery CTA: After therapy and follow-up, the floating thrombus in the right ventricle was verified to be the cause of the absence of any evident aberrant density shadow in the pulmonary artery CTA (Fig. 1C). Additionally, more follow-up results are shown in Figure S1, Supplemental Digital Content, http://links.lww.com/MD/O289, as evidence further supported the diagnosis of thrombosis in this case.

### 2.2. Case 2

**Figure 2.**
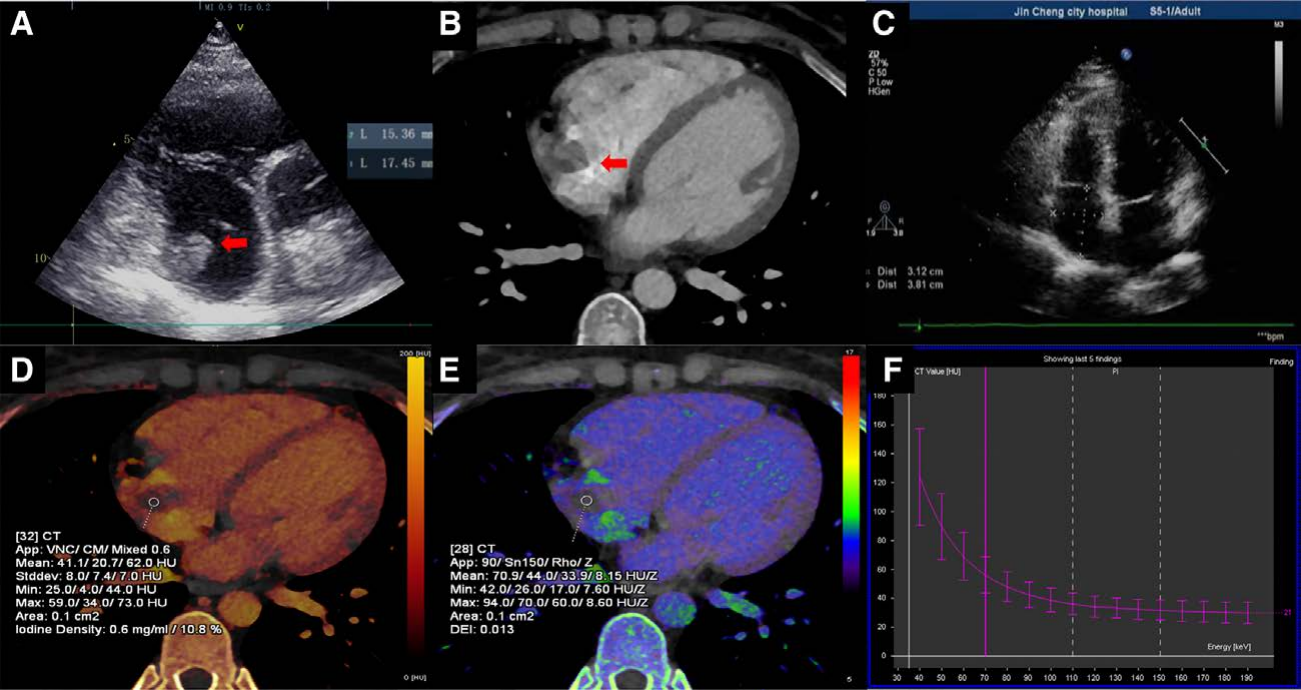
Case 2. The images show a thrombus in the right atrium. Echocardiography showed an isoechoic mass in the right atrium, measuring about 15.36 × 17.45 mm (A); CT enhancement scan indicated a classically round filling defect in the right atrium (B); The arterial-phase iodine map showed the arterial-phase iodine concentration in the lesion of the patient in the present case was 0.6 mg/mL (D); The effective atomic number map shown the lesion of the patient in the present case had a Zeff of 8.15 HU/Z (E); and the energy spectrum curve was schematic, with λ_Hu_ being valued as 1.1 (E). After 2 months of anticoagulation therapy, the echocardiogram was repeated, and no abnormal echoes were seen in the chambers of the heart (C).

The patient, a 39-year-old female, underwent echocardiography and dual-energy CT scan during chemotherapy for breast malignancy. The echocardiography showed an isoechoic mass in the right atrium, measuring about 15.36 mm × 17.45 mm (Fig. 2A); the CT enhancement scan indicated a classically round filling defect in the right atrium (Fig. 2B); the arterial-phase iodine map demonstrated arterial-phase iodine concentration in the lesion of the patient in the present case was 0.6 mg/mL (Fig. 2D); the effective atomic number map showed the lesion of the patient in the present case had a Zeff of 8.15 HU/Z (Fig. 2E); and the energy spectrum curve was schematic, with λHu being valued as 1.1 (Fig. 2F). After 2 months of anticoagulation therapy, the echocardiogram was repeated, and no abnormal echoes were seen in the chambers of the heart. Echocardiography was reviewed after 2 months of anticoagulation therapy, and no abnormal echoes were found in the chambers of the heart (Fig. 2C), which confirmed the right atrial thrombosis case by the follow-up of treatment. As shown in the follow-up results of ultrasound (Figure S2, Supplemental Digital Content, http://links.lww.com/MD/O289),the disappearance of the right atrial mass is considered evidence for diagnosing thrombus.

### 2.3. Case 3

**Figure 3.**
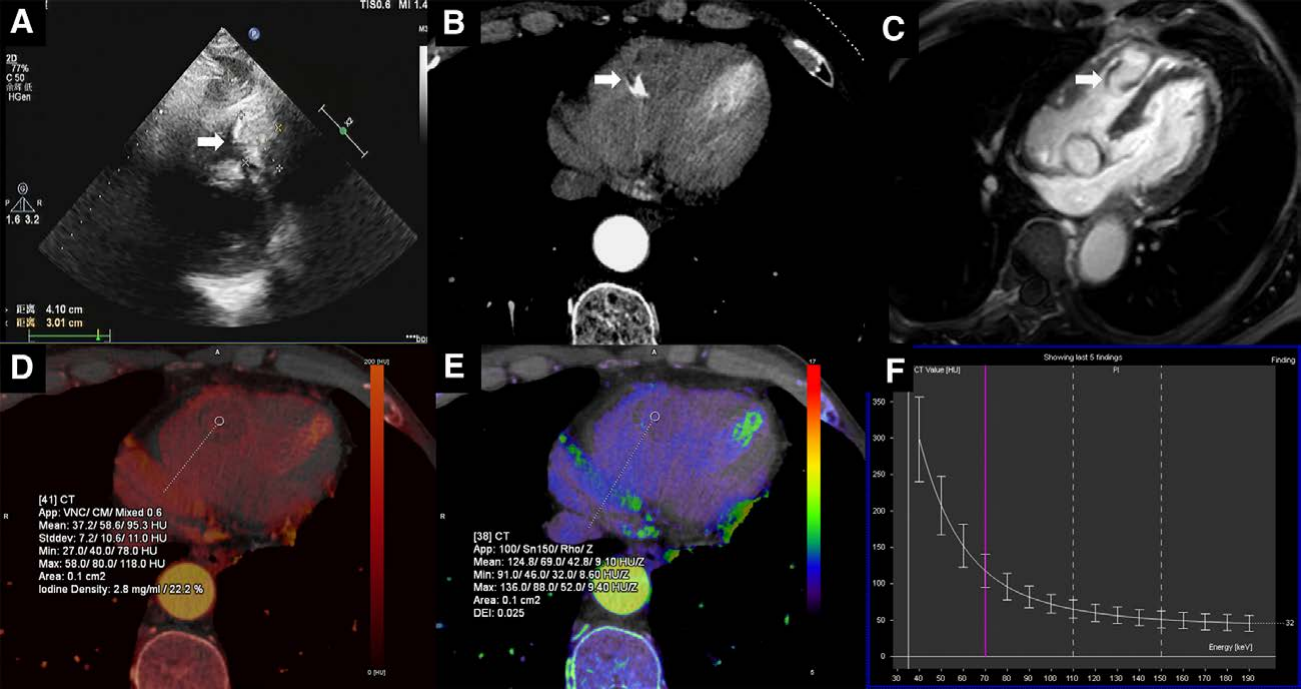
Case 3. The images show a myxoma in the right ventricle. Echocardiography, right ventricular free wall heterogeneous echogenic mass, lobulated, suggestive of right ventricular free wall mass (A); CT enhancement scan, right ventricle can be seen in a class of rounded low-density shadows, the edge of the arc-shaped calcification (B); MRI enhancement examination shows that the right ventricular free wall mass-like enhancement shadows, the edge of the visible line-like non-enhanced area, the lesion and the right ventricular free wall wide base is connected to the consideration of the possibility of myxoma (C). The arterial iodine concentration of the lesion was 2.8 mg/mL in the arterial iodine map (D); the effective atomic number map showed that the Zeff of the lesion was 9.10 HU/Z (E); and the λ_Hu_ of the energy spectrum curve showed that the λ_Hu_ was 2.9 (F).

The patient was a 67-year-old male who underwent echocardiography, dual-energy CT, and magnetic resonance during treatment for a benign tumor of the sigmoid colon. The echocardiography suggested a right ventricular free wall heterogeneous echogenic mass, which looked lobulated, and suggestive of right ventricular free wall mass (Fig. 3A). From the CT enhancement scan, the right ventricle could be seen in a class of rounded low-density shadows, the edge of the arc-shaped calcification (Fig. 3B); MRI enhancement examination showed that the right ventricular free wall mass-like enhancement shadows, the edge of the visible line-like non-enhanced area, the lesion and the right ventricular free wall wide base were connected to the consideration of the possibility of myxoma (Fig. 3C). The arterial iodine concentration of the lesion was 2.8 mg/mL in the arterial iodine map (Fig. 3D); the effective atomic number map showed that the Zeff of the lesion was 9.10 HU/Z (Fig. 3E); and λHu of the energy spectrum curve showed that the value of λHu was 2.9 (Fig. 3F). In addition, more detailed follow-up information is shown in Figure S3, Supplemental Digital Content, http://links.lww.com/MD/O289. After surgical resection, the patient was pathologically confirmed to have a cardiac myxoma in the right ventricle.

## 3. Discussion

Among reports of cardiac masses, the most common are cardiac myxoma and cardiac thrombosis. This case series provides new diagnostic ideas for the differentiation of myxoma from thrombus in rare parts of the heart.

In our reported cases, the first patient’s mass was located in the right ventricle, and it was relatively easy for us to diagnose it as a migratory thrombus in the deep veins of the lower extremities based on clinical symptoms such as chest tightness and dyspnea, abnormal clinical indicators such as D-2 polymer, and the morphology of the lesion as a striated cord. The second patient, with an insidious onset of the disease, and had a long-term intravenous catheter in the superior vena cava due to chemotherapy for breast malignancy. The mass in the right atrium was spherical and closely related to the intravenous catheter, so we assumed that it was an intravenous catheter-associated right atrial thrombus from an oncology patient, but because it was adjacent to the posterior wall of the right atrium and was fixed in position, and the size of the mass did not decrease significantly in the early stage of anticoagulation, it was difficult to identify it from the tumorous occupying lesions; In the third patient, the lesion was oval in shape, connected to the right ventricular free wall in a wide base, and curved calcified shadows were seen at the edge of the lesion, which demonstrated a filling defect similar to a cardiac thrombus on enhanced CT, and was indistinguishable from a thrombus on conventional CT except for the calcified features. Therefore, based on the previous literature^[5]^ that the mean iodine density of dual-energy CT scan quantitatively distinguish between cardiac myxoma and thrombus, and combined with dual-energy parameters such as slope of the energy spectrum curve and Zeff, which have the ability to separate substances and describe the composition of substances, we made detailed measurements and comparisons of the above 3 cases, and the results showed that the mean iodine density, the slope of the energy spectrum curve and Zeff of the cardiac myxoma were higher than that of the thrombus, and the mean iodine density of thrombus were 0.3 mg/mL, 0.6 mg/mL, and the slopes of spectral curves were 1.6 and 1.1, the Zeff were 8.34 HU/Z, 8.15 HU/Z, respectively; the mean iodine density of cardiac myxoma was 2.4 mg/mL, the slope of spectral curves was 2.9, and the Zeff was 9.10 HU/Z. We hypothesize that the mean iodine density ranges from 0 to 3 mg/mL, the slope of the energy spectrum ranges from 1 to 3,and the Zeff ranges from 8 to 9 HU/Z, and that there may be threshold values within these ranges that can be used to differentiate between cardiac myxoma and thrombus, and that the closer the measurements are to the lower limit, the greater the likelihood of a diagnosis of thrombus, and conversely the closer the measurements are to the upper limit, the greater the likelihood of a diagnosis of cardiac myxoma. In our results, the mean iodine density of both thrombus and cardiac myxoma were lower than those measured in previous studies, but the difference between the two was approximately the same as the difference measured in that study, and further verified that dual-energy CT iodine density has the ability to quantitatively differentiate between substances, and we analyzed that the diminished measurements may be related to differences in the method of contrast agent injection, scanning machines, and measurement methods. The differential diagnosis of dual-energy CT manifestations between cardiac thrombus and myxoma are shown in Table 2.

**Table 2:** The differential diagnosis of dual-energy CT manifestations between cardiac thrombus and myxoma

	Cardiac myxoma	Thrombus
Position	RV	RA, RV
Shape	Quasi-circular	Quasi-circular, striped
Tied attachment	(+)	(−)
Calcification	(+)	(−)
Iodine density (mg/mL)	>1	<1
Effective Atomic Number (HU/Z)	>9	<9
Spectrum curve slope	>2	<2

Transthoracic echocardiography clearly displays the size, morphology, and location of intracardiac occupations, allowing real-time observation of tumor oscillations, and is rapid, making it the imaging technique of choice for the detection of cardiac occupations.^[6]^ However, it lacks tissue specificity, making accurate diagnosis challenging when cardiac myxoma are visually similar to the echoes of a thrombus.^[7]^

MRI has greater soft-tissue resolution, supports multisequence and multiparameter imaging, and is the cardiac imaging technology recommended by the guidelines. Cardiac MRI first-pass perfusion and delayed enhancement scans have been shown to be critical for accurate cardiac tumors diagnosis. However, when myxoma is combined with extensive hemorrhage, the lack of contrast enhancement on delayed rolled imaging frequently makes it difficult to distinguish from thrombi.^[8]^ Furthermore, MRI is insensitive to the detection of calcification, which is a crucial indicator in distinguishing cardiac myxoma from thrombi and other space-occupying lesions.

In recent years, dual-energy CT technology has gained popularity for vascular imaging. The use of energy spectrum CT for vascular imaging can improve the visualization of contrast agents in blood vessels by scanning quickly at a low dose. It can do all-round reconstruction using raw data and detect coronary heart disease and pulmonary embolism in a single enhanced scan.^[9]^ Dual-energy CT can also post-process gathered data using the difference in material attenuation coefficients obtained at different energies. An initial study of the material composition and material separation can be realized by performing a multi-parameter qualitative and quantitative assessment of the lesion utilizing methods including energy spectrum curves, iodine density maps, and effective atomic number maps.^[10,11]^ The spectrum curve, which displays the variation in CT values of imaging tissues with X-ray photon energy (keV), is the curve produced by dual-energy CT. The slope of this curve can, to some extent, reflect the properties of tissues and lesions due to the different attenuation coefficients of various tissues with X-ray energy; this is useful in differentiating lesions from normal tissues; The most dependable post-processing technique is the iodine density map, which is more sensitive than regular CT to reflect variations in the tissue composition of lesions and can offer functional parameters and correctly quantitatively assess iodine in blood vessels or organs; Effective atomic number, or Zeff, is a color-coded picture that can be used to illustrate greater degrees of material differences and characterize the composition of substances based on various tissue atomic numbers.^[12]^ Dual-energy CT can be used to distinguish between cardiac myxoma and thrombi using quantitative analysis techniques, according to previous studies that evaluated the differences in mean CT attenuation density and mean iodine concentration between myxoma and thrombi using the Mann–Whitney *U* test.^[5,13]^ It hasn’t been documented yet, though, how the energy spectrum curve, iodine density map, and Zeff map are applied in tandem to the diagnosis of cardiac space-occupying lesions.

This study qualitatively and quantitatively evaluated thrombi and myxoma in uncommon areas of the heart using techniques such as a dual-energy CT energy spectrum curve, the iodine density map, and the effective atomic number map. The findings revealed that the slope of the energy spectrum curve, average iodine concentration, and effective atomic number of the thrombus were numerically similar, and all were lower than those of myxoma.

It should be noted that our study still has some limitations. First, the sample size was too small to validate the statistical significance of our measurements, and since cardiac tumors are a rare disease, recruiting a sufficient number of patients faced great difficulties; second, the patients with thrombus in the case series did not complete the MRI examination, which is the gold standard for detecting cardiac tumors, and of the 3 patients, only the patients with cardiac myxoma underwent MRI, making it impossible to compare the MRI imaging differences between thrombus and cardiac myxoma. Moreover, although dual-energy CT is a noninvasive modality, it involves a certain dose of radiation and is therefore not the best choice for detecting cardiac masses. Based on the cases we have reported, we believe that dual-energy CT may have a complementary role to play when encountering situations where MRI images cannot be obtained due to contraindications.

In the future, we will collect a sufficient number of cardiac myxoma and thrombus cases, improve the imaging methods as much as possible, verify the differences in the slope of the energy spectrum curve, the mean iodine density, and the presence of Zeff, and further clarify the value of combining multiple quantitative analyses, such as dual-energy CT energy spectrum curve, iodine density map, and Zeff, in the differentiation of cardiac mucinous tumors from thrombus.

## 4. Conclusion

We examined the dual-energy CT characteristics in 2 cases of intracardiac thrombus and one case of cardiac myxoma retrospectively. The findings demonstrated that in the 3 cases that were described, there were variations in the spectum curves’ slope, average iodine density, and Zeff between intracardiac thrombus and tumor. We believe that combining numerous quantitative analytic methods, such as the iodine density map, Zeff, and dual energy CT energy spectrum curve, can give an objective basis for the differential diagnosis of cardiac thrombus and myxoma based on the literature.

## Acknowlegments

The authors are very grateful to the patients for the providing their reports.

## Author contributions

**Data curation:** Yuxin Cui.

**Funding acquisition:** Yunbing Chen.

**Project administration:** Yunbing Chen.

**Writing – original draft:** Yunting Wang.

**Writing – review & editing:** Yunbing Chen, Honglin Lan.

## Supplementary Material


