# Supplementary figures and images for: Differential diagnosis of thrombosis and myxoma in unusual heart positions by dual-energy CT multiple parameter imaging: A case series
Source: Medicine (Baltimore). 2025 Jan 24;104(4):e41303. doi: 10.1097/MD.0000000000041303 (PMC11771713; doi:10.1097/MD.0000000000041303)

Supplementary Figure S1

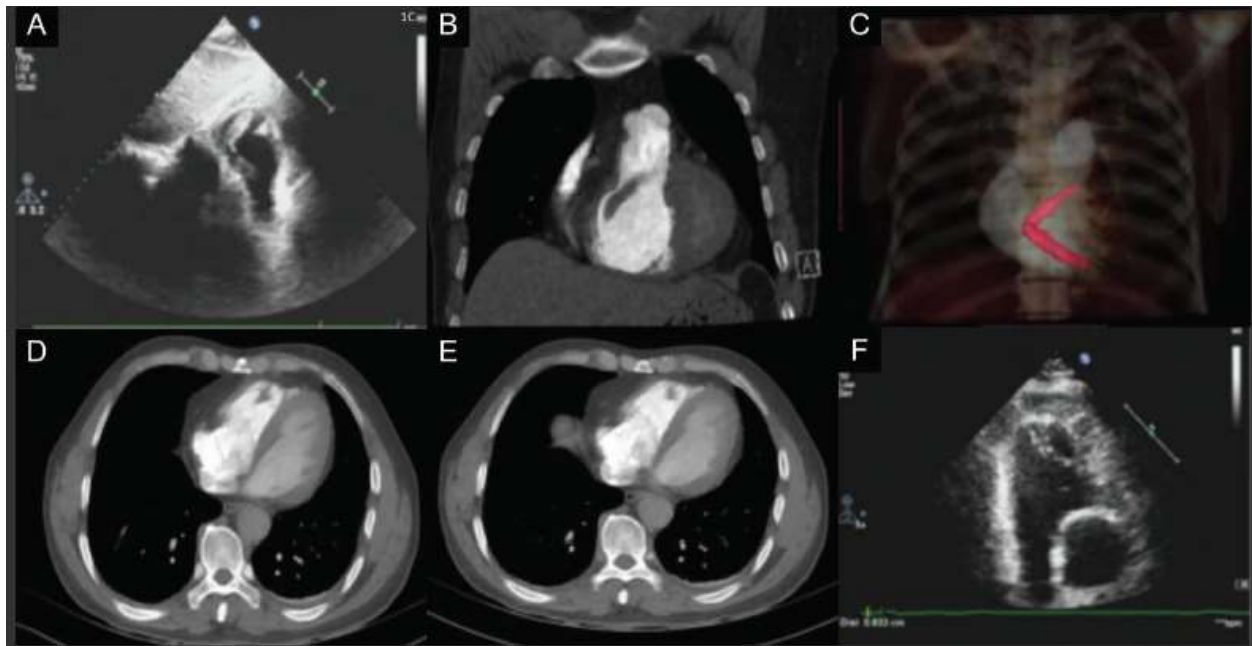

Supplementary Figure S2

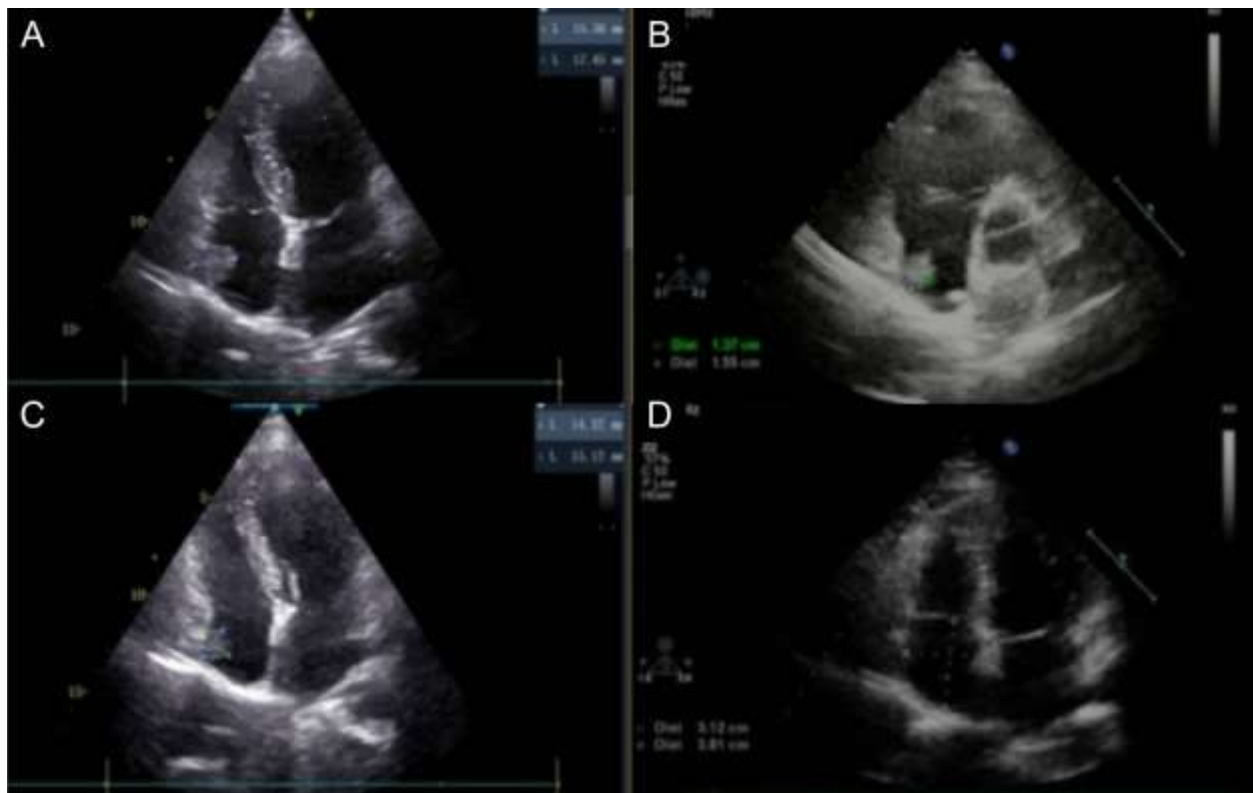

Supplementary Figure S3

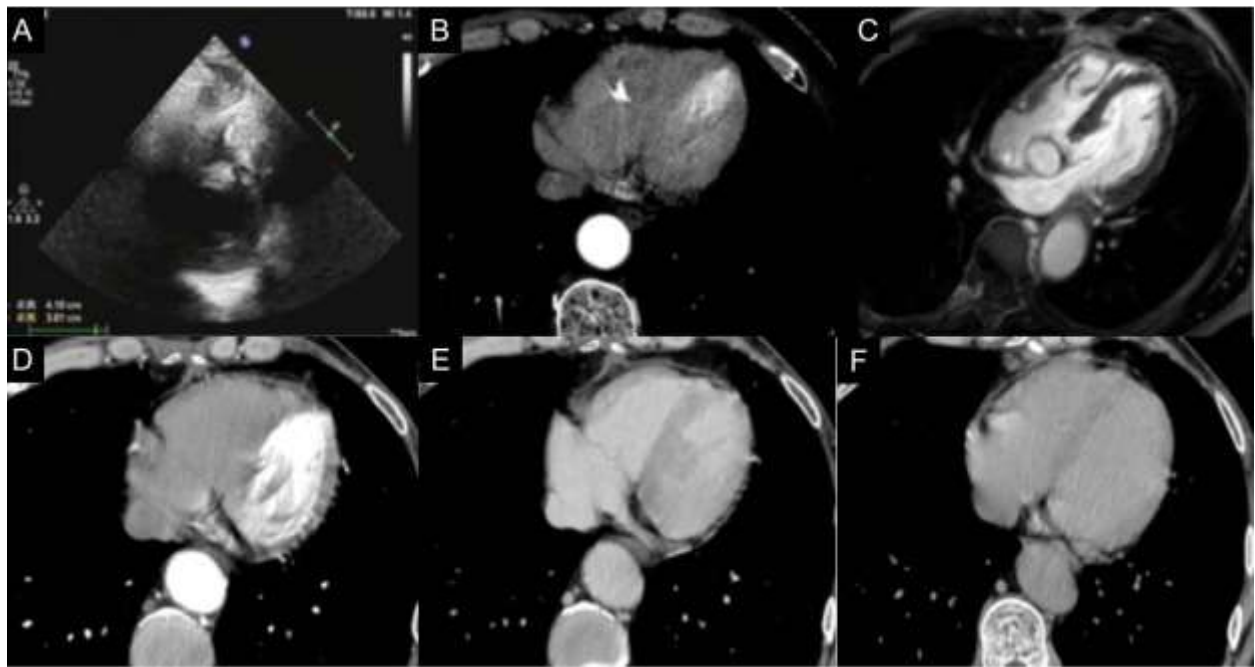

Supplement: Supplementary file 1 [file medi-104-e41303-s001.pdf]
